# National Trends in Suicide Among Asian American or Pacific Islander Youth

**DOI:** 10.1001/jamanetworkopen.2024.22744

**Published:** 2024-07-25

**Authors:** Brian TaeHyuk Keum, Seungbin Oh, Arielle H. Sheftall

**Affiliations:** 1 Department of Counseling, Developmental & Educational Psychology, Boston College, Chestnut Hill, Massachusetts; 2 Department of Psychiatry, Boston University School of Medicine, Boston, Massachusetts; 3 Department of Psychiatry, University of Rochester Medical Center, Rochester, New York

## Abstract

This cross-sectional study examines trends in suicide rates among Asian American or Pacific Islander youth by sex from 1999 to 2021.

## Introduction

Trends from the National Violent Death Reporting System between 2018 and 2019 suggest that while age-adjusted suicide rates decreased for White individuals, they increased for Asian American or Pacific Islander youths.^1^ This increase calls for an exploration of the suicide trend in this group, particularly between female and male youths given distinct gendered racial suicide risk factors.^2^ Thus, we examined suicide rates among Asian American or Pacific Islander youth by sex from 1999 to 2021.

## Methods

This cross-sectional study was exempt from institutional review board approval and informed consent given the use of public mortality data, per Common Rule guidelines. We followed the STROBE guideline.

We examined data from 1999 to 2021 (National Center for Health Statistics final multiple cause-of-death files through WISQARS^3^) for Asian American or Pacific Islander youth aged 10 to 19 years across all ethnicities (Hispanic or Latino, not Hispanic or Latino, and not stated) who died by suicide. Suicide deaths were determined through the *ICD-10* underlying cause-of-death codes U03, X60-X84, and Y87.0. Trend analysis was performed using Joinpoint regression (version 5.0.2; National Cancer Institute) to assess annual percent changes (APCs) in suicide rates, with statistical significance determined through weighted least-squares regression and a grid search algorithm (α level of .05).

## Results

**Figure 1.**
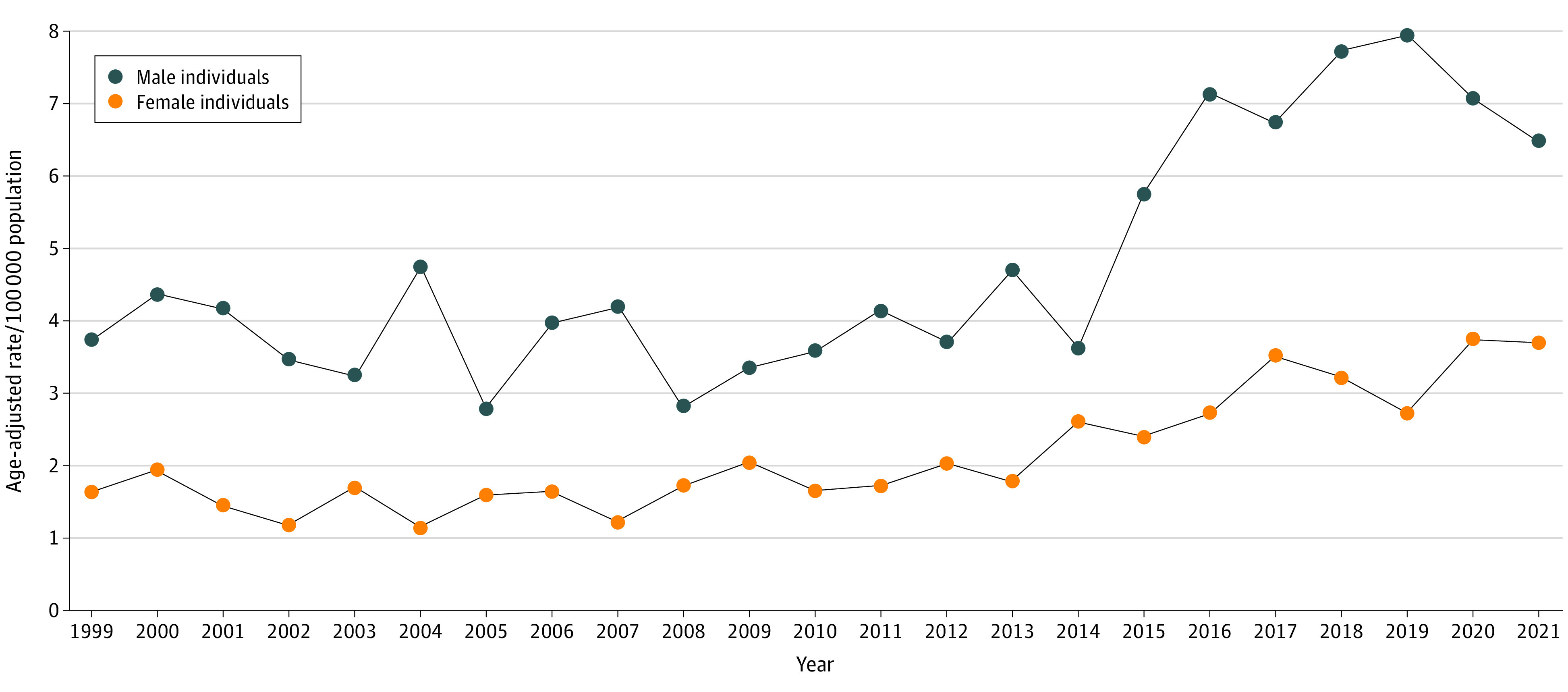
Age-Adjusted Suicide Rates Among Asian American or Pacific Islander Youths Aged 10 to 19 Years by Sex in the US, 1999-2021

This analysis included 1880 Asian American or Pacific Islander youths who died by suicide between 1999 and 2021. Individual-level demographics cannot be reported as the database only provides population estimates. The age-adjusted suicide rate (Figure 1) increased by 72% for male youths (from 3.76 to 6.49 per 100 000 individuals) and 125% for female youths (from 1.65 to 3.72 per 100 000 individuals) from 1999 through 2021. Suicide rates among male youths peaked in 2019 (7.96 per 100 000 individuals) and for female youths in 2020 (3.76 per 100 000 individuals).

**Figure 2.**
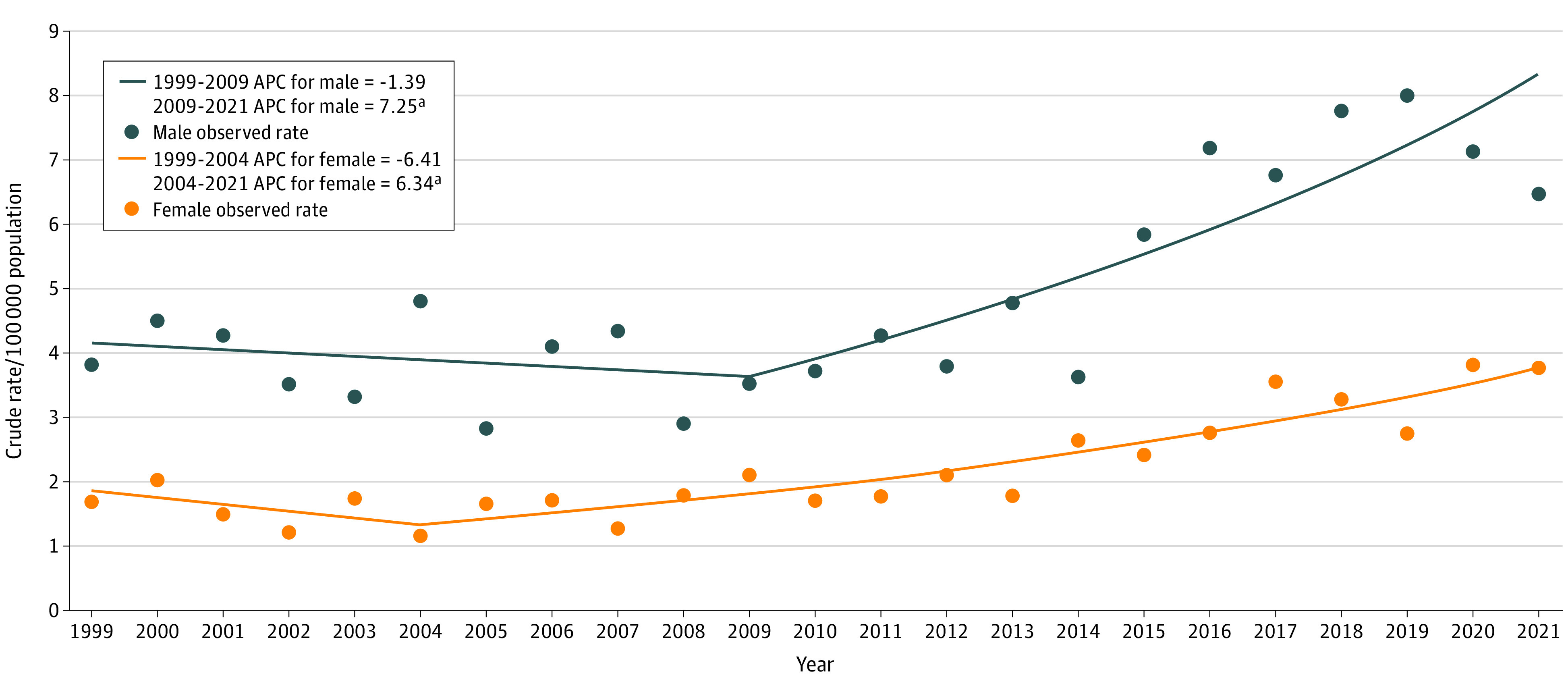
Joinpoint Regression Annual Percent Change (APC) in Suicide Rates by Sex, 1999-2021 ^a^APC is significantly different from 0 at α = .05.

Figure 2 displays shifts in suicide rates. For males, after a declining trend from 1999 to 2009 at an APC of −1.39% per year (95% CI, −6.13% to 3.59%; *P* = .56), a significant increasing trend emerged from 2009 to 2021 with an APC of 7.25% per year (95% CI, 4.25% to 10.33%; *P* < .001). For females, after a declining trend from 1999 to 2004 at an APC of −6.41% per year (95% CI, −17.34% to 5.95%; *P* = .28), a significant increasing trend was observed from 2004 to 2021, with an APC of 6.34% per year (95% CI, 4.81% to 7.89%; *P* < .001).

## Discussion

The sharp increase in suicide rates among Asian American or Pacific Islander male youths since 2009 follows the elevated suicide ideation reported in this group nationally.^4^ An upward shift among female youths in 2004 coincides with peak rates of suicide plans and attempts reported in this group nationally.^4^ Possible explanations include economic hardship in Asian American or Pacific Islander families during the Great Recession (2007-2009) and cyberbullying with the rise of social media platforms.^5,6^ Mental health struggles, including suicide ideation and behaviors of Asian American or Pacific Islander male family leaders during the recession, may have been a vicarious family-related risk factor for male youths. Social media has proliferated online sexism and racism against Asian women, which may have been internalized as an interpersonal risk factor for suicide for female youths.^2^ Factors such as intersectional discrimination (eg, compounding of racism and sexism) may contribute to the greater increase in suicide rates among females than males.^2^ The distinct increase in both groups calls for an examination of culturally informed etiology and trajectory of suicide risks with an eye toward gendered and racialized factors (eg, gendered racism and gender norms).^2^ Findings are limited by potential misclassification of suicide deaths in mortality records. Our findings may not be generalizable across distinct ethnic subgroups within the Asian American or Pacific Islander community, as the data aggregated these individuals into a single racial category.

## Notes

Supplement.
Data Sharing Statement
