# Supplementary material for: National Trends in Suicide Among Asian American or Pacific Islander Youth
Source: JAMA Netw Open. 2024 Jul 25;7(7):e2422744. doi: 10.1001/jamanetworkopen.2024.22744 (PMC11273234; doi:10.1001/jamanetworkopen.2024.22744)
Supplement: Supplement. — Data Sharing Statement [file jamanetwopen-e2422744-s001.pdf]

Data Sharing Statement

Data
Data available: No

Additional Information
Explanation for why data not available: Data may be shared upon request. The current study used publicly available data from the National Center for Health Statistics final multiple cause-of-death files through WISQARS. (Web-based Injury Statistics Query and Reporting System).
